# Nicotinic Restoration of Excitatory Neuroplasticity Is Linked to Improved Implicit Motor Learning Skills in Deprived Smokers

**DOI:** 10.3389/fneur.2018.00367

**Published:** 2018-05-28

**Authors:** Jessica Grundey, Nivethida Thirugnasambandam, Rosa Amu, Walter Paulus, Michael A. Nitsche

**Affiliations:** ^1^Clinical Neurophysiology, Georg-August-Universität Göttingen, Göttingen, Germany; ^2^Forschungsbereich Psychologie und Neurowissenschaften, Leibniz Research Centre for Working Environment and Human Factors (LG), Dortmund, Germany

**Keywords:** neuroplasticity, nicotine, non-invasive brain stimulation, cognition, smokers

## Abstract

Nicotine has been shown to modulate neuroplasticity, cognition, and learning processes in smokers and non-smokers. A possible mechanism for its effect on learning and memory formation is its impact on long-term depression and long-term potentiation (LTP). Nicotine abstinence in smokers is often correlated with impaired cognitive performance. As neuroplasticity is closely connected to learning and memory formation, we aimed to explore the effect of nicotine spray administration in deprived smokers on paired-associative stimulation (PAS25)-induced neuroplasticity and on performance of the serial reaction time task (SRTT), a sequential motor learning paradigm. Deprived smokers (*n* = 12) under placebo medication displayed reduced excitatory neuroplasticity induced by PAS25. Plasticity was restored by nicotine spray administration. Likewise, SRTT-performance improved after nicotine spray administration compared to placebo administration (*n* = 19). The results indicate a restitutional effect of nicotine spray in deprived smokers on both: LTP-like neuroplasticity and motor learning. These results present a possible explanation for persistence of nicotine addiction and probability of relapse.

## Introduction

Nicotine is the main psychoactive component of tobacco and responsible for its addictive properties (1). It modifies brain physiology *via* its interaction with nicotinic acetylcholine receptors (nAChR) (2). Especially, the alpha7- and the alpha4beta2-receptors are of major importance for brain physiology and cognition. Studies in animals and humans have shown improvements in attention, working, and episodic memory induced *via* application of nicotine (3–5). The physiological foundation of these functional effects of nicotine is still unclear but might be linked to calcium-channel properties of respective nAChRs. Intracellular Ca^2+^ concentration is relevant for the induction and modulation of neuroplasticity (6–8), including long-term potentiation (LTP) (9). Most studies exploring physiological and cognitive effects of nicotine so far have been performed in cell and animal models. With non-invasive brain stimulation techniques like transcranial direct current stimulation (tDCS) and paired associative stimulation (PAS), cortical neuroplasticity can be examined in humans (10–12). Both protocols induce NMDA-receptor and calcium channel-dependent plasticity that are similar to long-term depression and LTP (13–15). As tDCS non-selectively affects neuronal populations, it is referred to as non-focal plasticity inducing technique (16, 17), while PAS affects specifically activated synapses between sensory and motor neurons and thus induces a more focal, synapse-specific neuroplasticity (18). Its timing-specificity is furthermore related to spike timing-dependent plasticity, an animal slice model of plasticity thought to be closely related to memory formation (19). Former studies of our group have examined the effect of nicotine patch on LTP-like focal and non-focal neuroplasticity in deprived smokers. During nicotine deprivation, focal and non-focal excitatory plasticity was abolished, while nicotine patch administration partially increased motor cortex excitability to motor-evoked potential (MEP)-levels of non-smoking subjects (20–22). Thus in smokers, administration of nicotine patch restores compromised LTP-like plasticity. Regarding cognitive effects of nicotine, our earlier studies have shown that nicotine patch administration in deprived smokers has likewise restitutional effects of working memory performance, implicit motor learning, and attentional processes (23, 24). Nicotine patch and nicotine spray differ in terms of pharmacokinetics. Transdermal patches contain a large quantity of nicotine that has a membrane-limited delivery rate and can thus sustain stable nicotine levels over extended periods of time (25). Nicotine spray on the other hand has a sharp rise and a slower decline in plasma levels, thus mimicking the pattern associated with smoking a cigarette (26). With those differences in mind, we aimed to investigate the effect of nicotine spray on PAS-25 induced LTP-like motor cortex plasticity and Serial Reaction Time Task (SRTT) performance in deprived smokers. We chose the SRTT task, because it is a well-introduced instrument to explore motor learning in humans (27), which involves the primary motor cortex (28) and can distinguish between general motor skill learning (GMS) and sequence specific learning (SS) (29). GMS learning hereby indicates the acquisition of general performance, while SS indicates the learning of the repeated motor sequence. Given the fact that learning functions are closely linked to LTP-like plasticity, both neuroplasticity and cognitive performance were tested. Based on the pharmacology and plasma levels reached with both application forms (nicotine spray and nicotine patch) (8–9 ng/ml), we hypothesized that nicotine spray is able to restore lacking LTP-like plasticity in deprived smokers and improves motor learning, similar to the impact of nicotine patch on brain physiology and cognitive performance.

## Materials and Methods

### Subjects

Altogether, 31 healthy smokers participated and completed this study (12 in the PAS-25 experiment and 19 in the SRTT task). Table 1 displays the demographic characteristics of the subjects in terms of age, gender, and Fagerstrøm scale for nicotine dependence (30). Participants were recruited among students of the University of Goettingen and gave written informed consent prior to participation in the study. All participants had to be abstinent from nicotine 6 h prior to the experiments. Exclusion criteria, as obtained in a clinical interview and medical examination, were cardiac pacemaker, metal implants in the head, age younger than 18 and older than 50 years, current intake of any medication, current or history of neurological, psychiatric or medical disease, pregnancy or breastfeeding, current or previous drug (other than nicotine) or alcohol abuse and participation in other trials during the past 8 weeks. The experiments were approved by the Local Ethics Committee and conformed to the principles laid down in the Declaration of Helsinki. Allocations of the subjects to the respective experimental conditions as well as order of sessions were randomized.

**Table 1:** Subject data.

	Paired associative stimulation	Serial reaction time task	Statistics
Number of subjects	12	19	n.a.
Gender^a^	5 f/7 m	9 f/10 m	*p* = 0.756
Age^b^	26.1 ± 2.6	26.4 ± 2.9	*p* = 0.821
Fagerstrøm^b^	2.9 ± 1.3	3.4 ± 1.8	*p* = 0.410
Cigarettes^b^	15.5 ± 3.6	14.3 ± 4.7	*p* = 0.517
Duration^b^	8.0 ± 2.3	7.7 ± 2.2	*p* = 0.798

*Table 1 describes the subject data in terms of age, gender, Fagerstrøm nicotine dependency scale, cigarettes per day, and duration of nicotine addiction. There were no significant differences between both groups*.

*f, female; m, male; n.a., not applicable; p, p-value*.

*^a^Chi^2^-test*.

*^b^Paired t-test*.

### Paired Associative Stimulation

Twelve subjects participated in the PAS experiment (PAS-25). Hereby, a peripheral electrical pulse over the right ulnar nerve at wrist level was delivered by a Digitimer D185 multipulse stimulator (Digitimer, Welwyn Garden City, UK) and followed by a single transcranial magnetic stimulation (TMS) pulse over the motor cortex representation of the abductor digiti minimi muscle (ADM). The peripheral nerve stimulation was set to an intensity of 300% above sensory perceptual threshold; the applied TMS-pulse to a stimulator output resulting in MEPs amplitudes of approximately 1 mV (“baseline intensity,” see description in Section “Assessing Motor Cortex Excitability”). The paired pulses were repeated 90 times at a frequency of 0.05 Hz. The interstimulus interval of 25 ms, which was applied in this study, induces excitatory long-lasting excitability changes (12, 31). The participants were instructed to count the number of pulses they received at their wrist throughout the whole stimulation duration to guarantee sufficient attention to the procedure, which has been shown to be crucial to obtain the intended effects (32). The PAS protocol was combined with either nicotine or placebo spray for each subject in different experimental sessions.

### Assessing Motor Cortex Excitability

**Figure 1:**
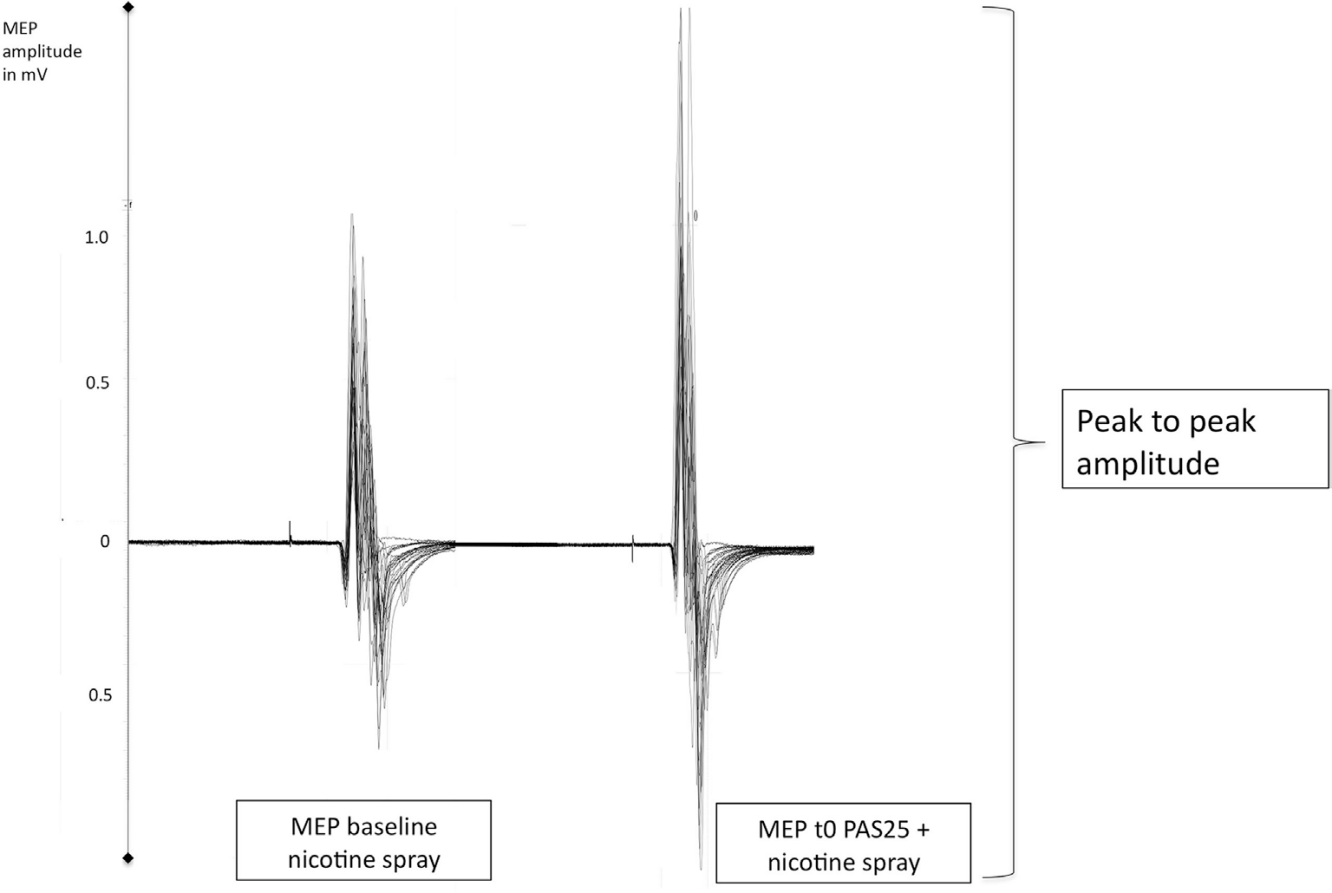
Displays original motor-evoked potential (MEP)-recordings of a smoking individual after administration of nicotine spray (baseline) and then directly after the PAS25-protocoll (t0). PAS25 enhances the transcranial magnetic stimulation-induced MEP-amplitudes as measured by the peak to peak amplitude and read off on the *y*-axis (MEP-values). mV, millivolt; t0, first timepoint after PAS25-protocoll.

Transcranial magnetic stimulation-elicited MEPs were recorded to measure excitability changes of the representional motor cortical area of the right ADM. Single pulse TMS was conducted by a Magstim 200 magnetic stimulator (Magstim Company, Whitland, Dyfed, UK) at a frequency of 0.25 Hz with a figure of eight-shaped coil (diameter of one winding 70 mm; peak magnetic field, 2.2 T). The coil was held tangentially to the scalp at an angle of 45° to the sagittal plane with a coil handle pointing laterally and posterior. This induced a posterior–anterior current flow in the brain at an angle that optimally activates the corticospinal system monosynaptically (33). Surface EMG was recorded from the right ADM with Ag–AgCl electrodes in a belly tendon montage. The optimal position was defined as the site where stimulation resulted consistently in the largest MEPs and then marked with black ink. The signals were amplified and filtered with a time constant of 10 ms and a low-pass filter of 2.5 kHz, then digitized at an analog-to-digital rate of 5 kHz, and further relayed into a laboratory computer using the Signal software and CED 1401 hardware (Cambridge Electronic Design). The intensity was adjusted to elicit, on average, baseline MEPs of 1 mV peak-to-peak amplitude, and was kept constant for the post-intervention stimulation (see also Figure 1). Changes of the mean MEP amplitude over time reflect alterations of motor cortex excitability.

### Serial Reaction Time Task

**Figure 2:**
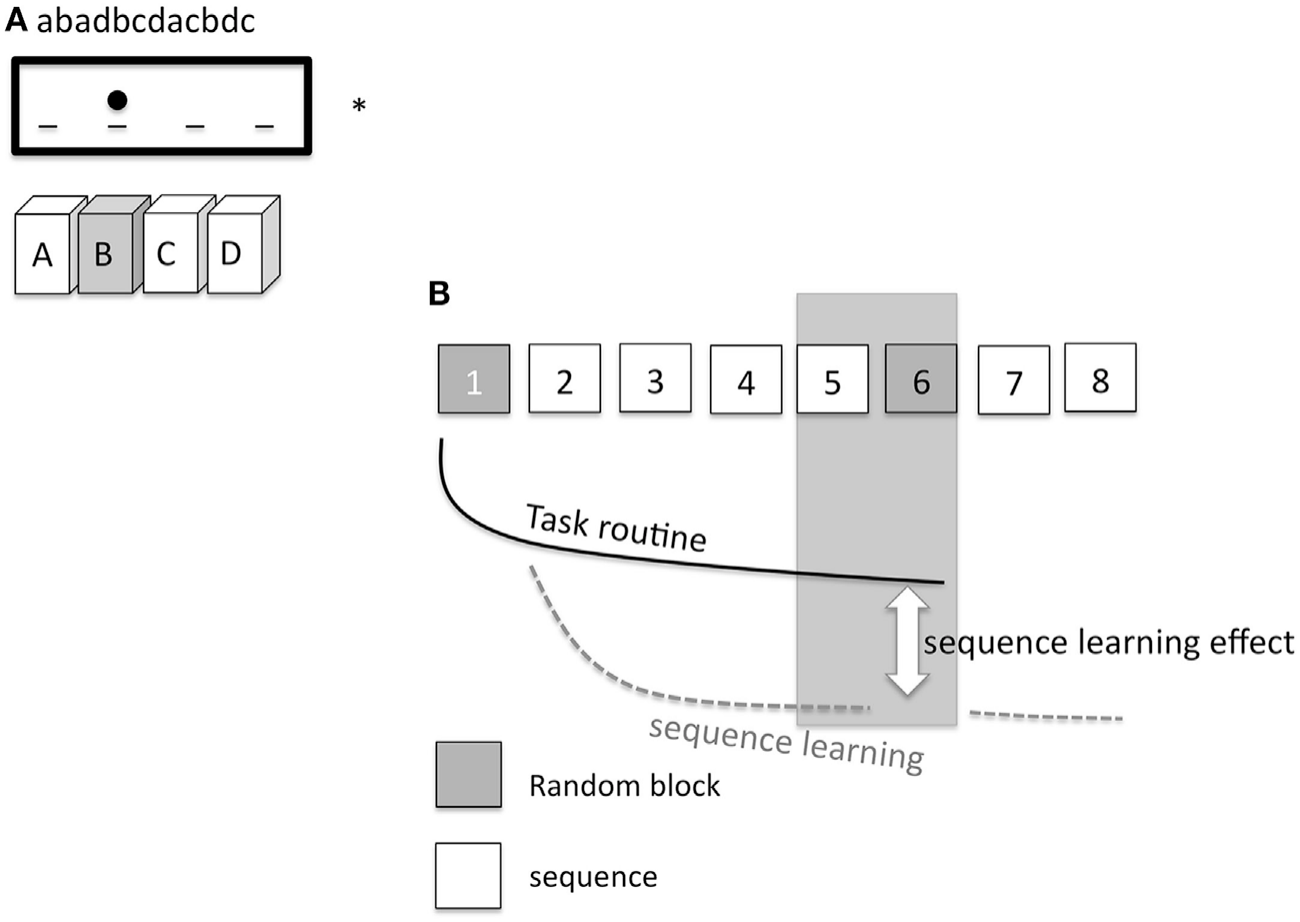
**(A)** Displays the setup of the serial reaction time task (SRTT). On a computer screen (*) the elements of the sequence (abadbcdacbdc) are shown consecutively and appear as dots at a specific position. A dot at the second position of the screen means that the corresponding second button on the pad needs to be pushed [gray filled box with the letter (B)]. The dot at the third position means that the subject has to push the third button (C), etc. **(B)** shows the specific SRTT-procedure. The procedure consists of 8 blocks. Block 1 and 6 (gray) replay a random order of dots (pseudorandomized sequence). Hereby, the dots are presented equally frequently in each position and never in the same position in two subsequent trials. In blocks 2–5 and 7–8, the same sequence of 12 dot positions is repeated 10 times (white squares). The black line signifies task routine [decrease of reaction time (RT), GMS], the dotted gray line shows the sequence learning curve. The difference between the task routine curve and the sequence learning curve determines the sequence learning effect (RT difference between block 5 and 6, SS).

Nineteen subjects participated in the SRTT experiment. Participants were seated in front of a computer monitor at eye level and asked to respond to a visual cue as quickly and accurately as possible by pressing the appropriate button on a keyboard. A response pad with four buttons (numbered 1–4) was placed in front of the subjects, and they were instructed to push each button with a different finger of the right hand (index finger for button 1, middle finger for button 2, ring finger for button 3, and little finger for button 4). A dot appeared in one of four positions, horizontally spaced on a computer screen. Participants were instructed to press the key corresponding to the position of the dot as fast as possible (Figure 2A). The learning test consisted of 8 blocks of 120 trials in each block. In blocks 1 and 6, the sequence of dots followed a pseudorandom order; here, dots were presented equally frequently in each position and never in the same position in two subsequent trials. In blocks 2 to 5 as well as in blocks 7 and 8, the same 12-trial sequence of dot positions was repeated 10 times (e.g., abadbcdacbdc; see also Figure 2B). Sequence-specific (SS) as well as general motor skill learning (GMS) results in improved performance during the whole course of the task, as mentioned above (24, 34). Differences in performance between block 5 and random block 6 represent a measure of sequence-specific motor learning only, because GMS is equivalent in both blocks (35). Subjects were not told about the repeating sequence but asked after the last block of each session if they were aware of a repeating sequence. Four versions of SRTT were generated and applied in a randomized order, so that every subject encountered each version only once to exclude interference effects. Performance level was evaluated *via* reaction time (RT) and error rates (ER) of each subject and in each condition.

### Pharmacological Intervention

PAS25-induced excitability changes and SRTT-performance were measured in different groups of participants. Twelve subjects participated in the PAS25- and nineteen subjects in the SRTT-experiment. Two sessions were carried out for each subject in both groups in randomized order. Inhalative nicotine spray contained either nicotine (10 mg/ml) or inactive placebo. The nicotinic nasal spray was administered in a cumulative dose of 1 mg nicotine (Nicorette^®^ Nasal Spray, McNeil Products, UK) to all subjects in combination with either the PAS25 intervention or SRTT performance. The rise time of nicotine by nasal spray in venous blood levels is close to nicotine blood levels delivered by cigarettes (36) with plasma peak levels after 5–10 min. Interventions (SRTT and PAS) were performed after 10 min to obtain effects of peak dose concentration. Side effects were coughing, sneezing, throat irritation, and dizziness, as described in prior clinical trials (37). Symptoms subsided rapidly within minutes.

### Course of the Experiment

#### Paired Associative Stimulation

Subjects were seated comfortably in a reclined chair with head and armrests and were asked to relax completely. The EMG electrodes were placed at the right ADM as described in the Section “Assessing Motor Cortex Excitability.” The exact position was marked with a pen. Then TMS was applied over the left representional area of the right ADM to determine the spot with the consistently highest MEPs in the resting ADM. This spot was also marked with a waterproof pen. TMS-intensity was set to elicit MEP amplitudes of 1 mA (S1mV). Twenty MEPs were recorded at this stimulus intensity and the mean was calculated and determined as baseline. Then nicotine nasal spray, or respective placebo spray was administered. 10 min later, after side effects like coughing and sneezing had subsided, and nicotine peak-dose concentration was achieved, MEP-amplitudes were controlled and adjusted if necessary. Then the PAS25-protocol was administered, followed by immediate recording of at least 20 MEPs at the time points of 0, 5, 10, 15, 20, 25, 30, 60, 90, and 120 min after intervention (Figure 1). Sessions were conducted in randomized order and with a minimal intersession interval of 1 week.

#### Serial Reaction Time Task

**Figure 3:**
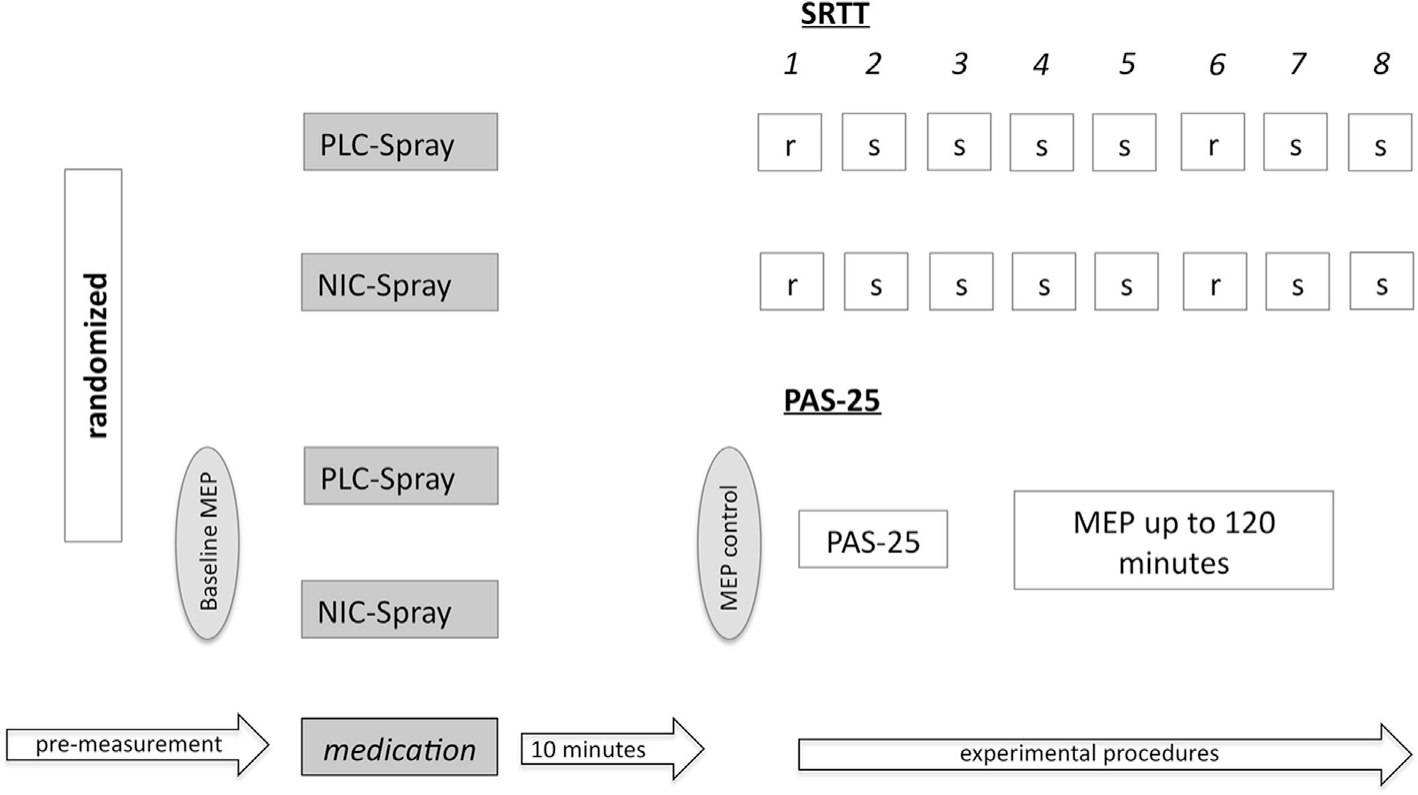
Shows the experimental setup of the serial reaction time task (SRTT) and the PAS-25 experiment. Subjects received either placebo or nicotine spray (PLC/NIC) in randomized order. After 10 min, the SRTT/PAS-25 protocol started. For the PAS-25 measurements, the MEP-baseline values were determined before and after drug administration, and then again monitored for up to 120 min post intervention. r = random stimuli; s = sequence stimuli; numbers 1–8 refer to the different blocks; MEP, motor-evoked potentials.

Before the beginning of SRTT performance, a short introduction and practice session was carried out with all participants to explain the rationale of task performance. Therefore, a test SRTT (programmed only for this reason) with one block was performed by the subjects before conduction of the main experiment. As in the PAS experiment, nicotine nasal spray or placebo spray respectively was then administered, and after 10 min, SRTT performance (as described in the Section “Serial Reaction Time Task”) was conducted. For course of experiments, see also Figure 3.

### Data Analysis and Statistics

#### PAS25

First, individual means of the 20 MEP amplitudes recorded at each time point were calculated. The post-intervention mean MEP amplitudes from each subject were then normalized to the respective individual mean baseline MEP-amplitude (quotient of post- versus pre-intervention MEP amplitude). The normalized mean MEP amplitudes from all subjects were then pooled together and the grand average across subjects for each time bin was calculated. A repeated measurement ANOVA was performed on the normalized data using MEP amplitude as the dependent variable (including all time points up to 120 min after stimulation). Drug (nicotine versus placebo), time points were included as within-subjects factors. The Mauchy test was performed to test for sphericity, and the Greenhouse–Geisser correction applied when necessary. Conditional on significant results of the ANOVA, we performed exploratory *post hoc* comparisons using Student’s *t-*tests (paired, two tailed, *p* < 0.05, not adjusted for multiple comparisons) to compare the MEP amplitude before and after the interventional brain stimulation in each condition and between drug conditions (nicotine/placebo) for each time point. A *p*-value of 0.05 was considered significant for all statistical analysis. Significances of differences in demographic factors were tested by one-way ANOVA and chi-square test for gender. Baseline MEP amplitudes and the maximum stimulator output percentage (% MSO) were analyzed before and after the interventional drug administration (nicotine spray and placebo) and between drug conditions (Student’s *t-*test, paired, two-tailed, *p* < 0.05). All data are expressed as mean ± SEM.

#### Serial Reaction Time Task

In each trial, RT was recorded from the appearance of the dot until the first button was pressed. To avoid artifacts, RTs <200 ms and above 3 SDs from the overall mean were excluded from further analysis, also the RTs of the incorrect responses. Mean RT was calculated for each participant, block, and medication condition separately. Furthermore, the SD of response times for each subject in every block was calculated as an index of variability of response times. Additionally, for each block, subject, and medication condition, the number of incorrect responses (error rates; ER) was calculated. Statistical analysis (SPSS version 24.0) was performed with a repeated measurement ANOVA separately for absolute RT-values, variability, and ERs [within subject factors: condition (nicotine spray versus placebo spray) and time bin (blocks 1–8)]. The Mauchly Test was performed to test for sphericity and the Greenhouse–Geisser correction applied when necessary. RT differences between block 5 (pseudo randomized order of cues) and block 6 with a randomized order of dots were calculated for all groups and conditions separately (d5-6). This difference is defined as mostly pure index of sequential motor learning (SS), because task routine (GMS) level between block 5 and 6 is thought to be stable (14). A repeated measurement ANOVA for this RT difference (block 5 and 6) was further calculated [within subject factor: CONDITION (nicotine spray versus placebo spray)]. Conditional on significant results of the ANOVAs, we performed exploratory *post hoc* comparisons using Student’s *t*-tests (paired, two-tailed, *p* < 0.05, not adjusted for multiple comparisons), where we compared the respective differences between placebo and nicotine conditions separately (ER, absolute RTs, d5-6, variability) for each block. A level of significance <0.05 was considered significant for all statistical analysis. Based on the results of the respective *t*-tests, Cohen’s *d* as measures of effect sizes was calculated (see Table 2).

**Table 2:** Cohen’s *d* (paired *t*-test).

Serial reaction time task (SRTT)	Condition		Block	*T*-value	No.	Correlation in *r*	Cohen’s *d*
	plc vs. nicotine	RT	1	2.626	19	0.767	0.41
	plc vs. nicotine	RT	2	2.487	19	0.78	0.39
	plc vs. nicotine	RT	3	2.29	19	0.687	0.42
	plc vs. nicotine	RT	4	2.522	19	0.672	0.47
	plc vs. nicotine	RT	5	4.066	19	0.732	0.69
	plc vs. nicotine	RT	6	2.818	19	0.53	0.63
	plc vs. nicotine	RT	7	2.665	19	0.796	0.39
	plc vs. nicotine	RT	8	3.671	19	0.828	0.49
SRTT	plc vs. nicotine	Errors	1	0.21	19	0.823	0.03
	plc vs. nicotine	Errors	2	2.167	19	0.573	0.46
	plc vs. nicotine	Errors	3	2.082	19	0.561	0.45
	plc vs. nicotine	Errors	4	1.117	19	0.589	0.23
	plc vs. nicotine	Errors	5	−0.427	19	0.503	−0.10
	plc vs. nicotine	Errors	6	0.158	19	0.227	0.50
	plc vs. nicotine	Errors	7	1.563	19	0.698	0.28
	plc vs. nicotine	Errors	8	0.653	19	0.337	0.17
Paired associative stimulation	plc vs. nicotine	Timepoint	t0	−3.003	12	0.457	−0.90
	plc vs. nicotine	Timepoint	t5	−3.326	12	0.307	−1.13
	plc vs. nicotine	Timepoint	t10	−2.617	12	0.371	−0.85
	plc vs. nicotine	Timepoint	t15	−1.178	12	0.320	−0.38
	plc vs. nicotine	Timepoint	t20	−1.395	12	0.299	−0.48
	plc vs. nicotine	Timepoint	t25	−0.574	12	0.177	−0.21
	plc vs. nicotine	Timepoint	t30	−1.184	12	0.248	−0.42
	plc vs. nicotine	Timepoint	t60	−1.872	12	−0.534	−0.95
	plc vs. nicotine	Timepoint	t90	−0.282	12	−0.374	−0.14
	plc vs. nicotine	Timepoint	t120	−1.538	12	0.005	−0.63

## Results

All subjects tolerated the experiments and the pharmacological intervention well. Sneezing and coughing after inhalation of nicotine spray occurred in 28 of 31 subjects, but subsided quickly without interfering with the interventions. No significant group differences were found in terms of age, gender, and TMS-intensity to elicit an MEP of 1 mV (S1mV) before and after administration of nicotine spray (see Tables 1 and 3).

**Table 3:** Comparison of transcranial magnetic stimulation (TMS)-parameters.

Stimulation	Condition	TMS Parameter	Baseline 1	Baseline 2
PAS 25	Spray^a^	Motor-evoked potential (MEP)	1.01	1.04
		MSO%	47	47.83
	Placebo^a^	MEP	1.06	1.058
		MSO%	46.5	45.41

*Table 3 lists the mean MEP-amplitudes (MEP in millivolts) and mean stimulation output (MSO in %) before and after nicotine spray or placebo spray administration. There were no significant differences between both groups (before and after) and both conditions (nicotine spray versus placebo spray)*.

*^a^Student’s t-test, paired, two-tailed, p < 0.05*.

### Effect of Nicotine Spray on PAS-25 Induced Excitatory Plasticity in Smokers

**Figure 4:**
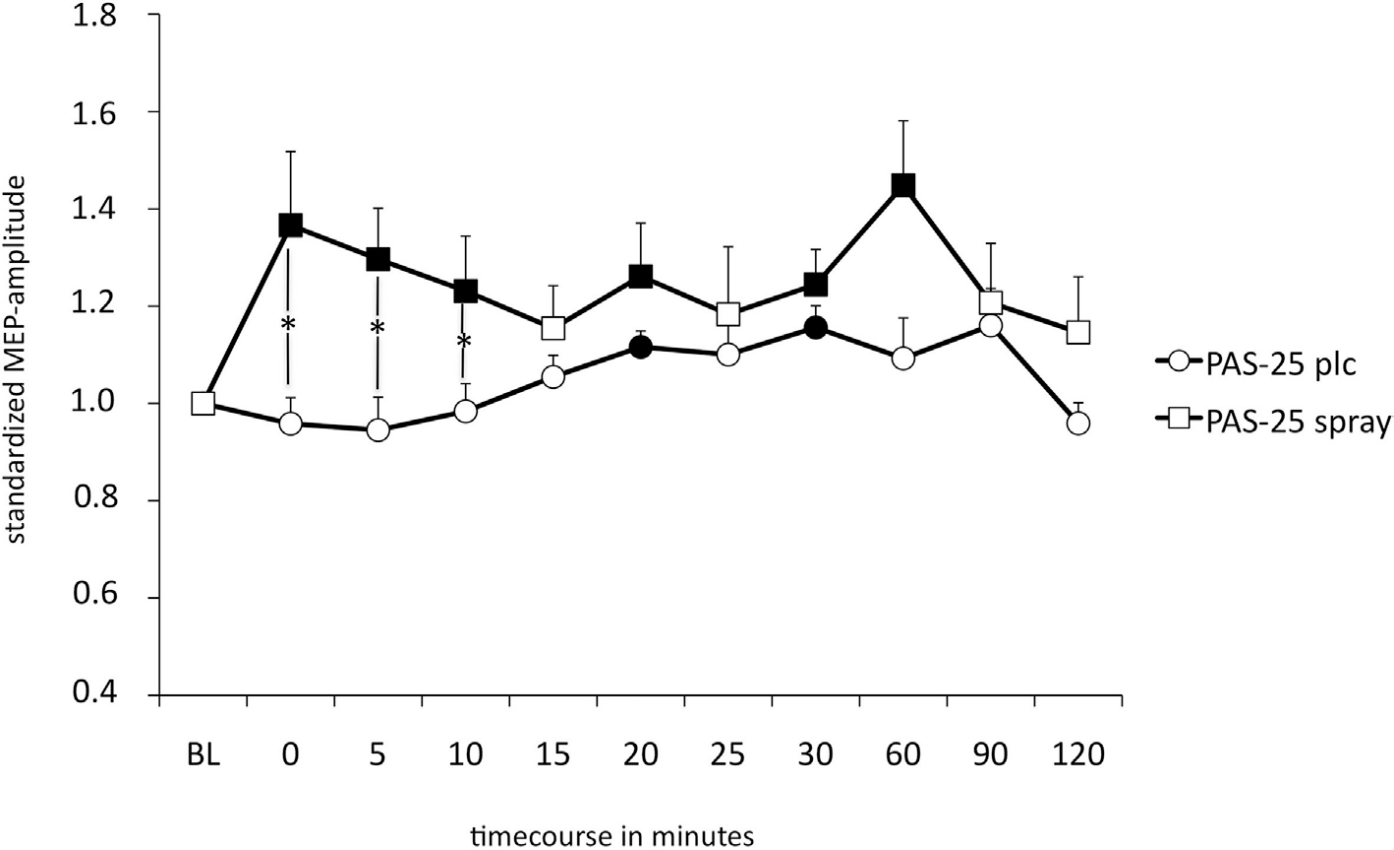
Displays the nicotinergic impact on paired associative stimulation (PAS)-induced excitatory neuroplasticity. Shown are graphs with motor-evoked potential (MEP)-standardized values on the *y*-axis plotted against different time points poststimulation on the *x*-axis. In smokers under placebo medication, PAS-25 did not enhance MEP amplitudes, while administration of nicotine spray leads to facilitatory after-effects lasting for up to 60 min after plasticity induction. Filled symbols indicate statistically significant deviations from baseline and asterisks indicate significant differences between the placebo medication and nicotine conditions (Student’s *t*-test, paired, two-tailed, *p* < 0.05). Abbreviations: BL, baseline; plc, placebo; spray, nicotine spray; error bars, SEM.

The repeated measurement ANOVA revealed a significant main effect of the within-subject factor “condition” (nicotine vs. placebo) but no significant interaction condition x time point (see also Table 4). As shown by the exploratory *post hoc t-*tests, smokers in a nicotine-deprived state after placebo drug administration did not show excitability alterations after the PAS-25 stimulation protocol. However, administration of nicotine spray partially re-established excitatory excitability changes after PAS-25 stimulation. Post interventional MEP amplitudes were significantly enhanced compared to baseline MEPs for the time bins 0, 5, 10, 20, 30, and 60 min after PAS (see Figure 4). The MEP amplitudes between the placebo and the nicotine spray conditions differed significantly for minutes 0, 5, and 10, which further indicate an enhancement of cortical excitability in deprived smokers after nicotine spray and PAS25-protocoll.

**Table 4:** ANOVA for SRTT/PAS-25.

Test	Parameters	Conditions	(df_N_, df_D_)	*F*-value	*p*-Value
SRTT	Absolute RT	Conditions	(1, 18)	13.979	0.002*
		Block	(7, 126)	16.549	0.001*
		Condition × block	(7, 126)	0.832	0.562
	Errors	Conditions	(1, 18)	1.333	0.263
		Block	(7, 126)	3.648	0.001*
		Condition × block	(7, 126)	1.401	0.211
	Variability	Conditions	(1, 18)	2.121	0.163
		Block	(7, 126)	2.607	0.015*
		Condition × block	(7, 126)	0.890	0.517
PAS-25	Motor-evoked potential-amplitudes	Condition	(1, 11)	11.607	0.006*
		Time points	(10, 110)	1.691	0.092
		Condition × time points	(10, 110)	1.490	0.152

Table 4 displays the *F*-value, the *p*-Value, and degrees of freedom of the repeated measurement ANOVA fort he SRTT and PAS-25 measurements. The ANOVA of the SRTT is divided in the different parameters: absolute RT, errors, and variability. Significant results (*p* < 0.05) are printed in bold and marked with *.

*df_N_, numerator degree of freedom; df_D_, demonitator degrees of freedom; SRTT, serial reaction time task; RT, reaction time*.

### Effect of Nicotine Spray on SRTT-Performance in Smokers

**Figure 5:**
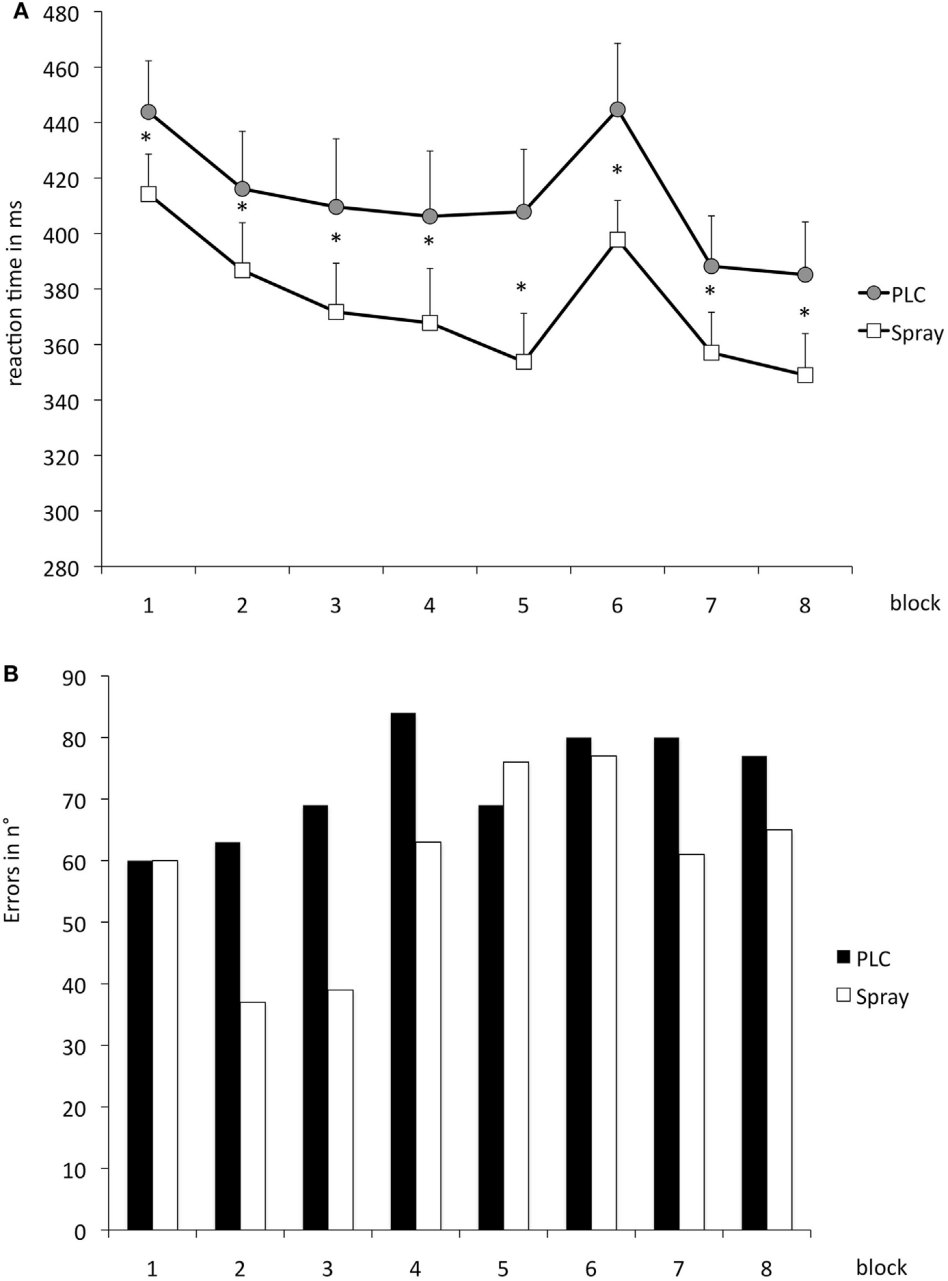
**(A)** Displays serial reaction time task performance after placebo and nicotine spray administration. Depicted are the absolute reaction times (RTs) (and SEs) and error rates **(B)** (summation) for both conditions. Block 1 and 6 present random stimuli, the remaining blocks contain the repeated sequence. Smokers during nicotine withdrawal under placebo spray administration display slower RTs in all blocks as compared to those in the nicotine spray condition. Abbreviations: ms, milliseconds; plc, placebo; spray, nicotine spray; *, significant differences in RTs between placebo spray and nicotine spray.

The repeated measurement ANOVA for absolute RT with the factors block and condition (placebo versus nicotine spray) resulted in significant results for the main factor “block” [*F*(1, 18) = 13.979; *p* = 0.02] and “condition” [*F*(7, 126) = 16.549; *p* < 0.001] (see also Table 4). *Post hoc* paired sample, two-tailed *t-*tests (placebo versus nicotine spray) revealed that the absolute RT of smokers after nicotine spray administration were significantly lower compared to the placebo administration in all blocks (Figure 5A). Implicit motor learning (or sequential skill learning; SS) that is evaluated by comparing the RT differences between block 5 and 6 (paired sample, two-tailed *t-*test), did not improve significantly between both pharmacological conditions (*p* < 0.582). For error rates and variability, the repeated measurement ANOVA revealed significant results for the main factor “block” [ER: *F*(7, 126) = 3.648; *p* < 0.001; variability *F*(7, 126) = 2.607; *p* = 0.015] due to a reduced number of errors and less variability during performance in the later blocks, but no significant main effect of nicotine or interactions (Figure 5B).

## Discussion

The results of the present study reveal that restitution of nicotine deprivation with nicotine spray in smokers has a prominent and rapid effect on PAS-25 induced excitatory neuroplasticity and SRTT performance. In smokers, nicotine deprivation prevents the induction of enhanced motor-cortex excitability measured by single pulse TMS and MEP-values, while administration of nicotine spray leads to enhanced MEP-values similar to MEP-values of non-smokers after PAS-25 (20, 21), and suggests hereby a restitutional effect of nicotine on LTP-like plasticity. In terms of SRTT-performance, administration of nicotine spray improved RTs in deprived smokers independent from the respective sequence condition, while error rates remain stable. Our data are in principal accordance with earlier studies of our group that have investigated the effects of long acting nicotine administration (nicotine patch) on PAS-induced excitability changes, memory, attention, and implicit motor learning in smokers (23). Similar to nicotine spray, nicotine patch administration was likewise able to restitute PAS-25 induced MEP-enhancements in deprived smokers (20). With regard to SRTT-performance, nicotine patch had a stronger impact on motor learning than nicotine spray. Nicotine patch not only improved RTs in general (in all blocks) but also the difference in RTs between block 5 and 6, which indicates a sequence specific learning effect (24).

### Proposed Mechanism of the Restitutional Nicotinic Effects on PAS-25-Induced Neuroplasticity and SRTT Performance

Glutamatergic plasticity, as accomplished by PAS, is thought to depend on the enhancement of intracellular calcium levels. Nicotine, as an agonist of nAChRs with calcium channel properties (both, α7 and α4β2 receptors), can increase intracellular calcium levels and transmitter release (38, 39). It has further been shown that chronic nicotine consumption can lead to desensitization of nAChRs in different areas of the brain (40, 41) and that the duration of the desensitization depends on the duration of nicotine exposure (42). Nicotine deprivation in chronic smokers might thus lead to a state with deficient calcium influx that does not exceed threshold levels necessary to enhance motor cortex excitability/LTP-like plasticity after PAS application. Re-application of nicotine with administration of nicotine spray might override the respective receptor desensitization and increase intracellular calcium influx to sufficient calcium levels that re-establish LTP-like neuroplasticity. In terms of cognitive data, our study is in principle accordance with earlier studies, which have shown deteriorated cognitive functions in nicotine-deprived smokers (23, 43). A partial restitution of cognitive performance was hereby achieved by re-administration of nicotine *via* nicotine patch. The effect of nicotine spray in our study was restricted to general motor skill learning alone, which is the main difference to an earlier study of our group, in which nicotine patch also improved sequence specific learning (24). Referring to these pro-cognitive nicotinic effects in deprived smokers, experiments in animals have already shown that hippocampal-dependent learning is influenced by nicotine based on the modulation of kinases and transcription factors (44). Nicotinic desensitization and/or upregulation of different AChRs may further alter learning processes (40, 45). The differences between nicotine spray and patch on motor learning might be due to the fact that pharmacokinetics between both applications differs. The dosages of nicotine spray and patch were chosen to induce comparable nicotinic blood levels [8–9 ng/ml (26, 46)]; still, nicotine administered by nicotine spray rises quickly and induces its peak plasma level after 10 min (36), while nicotine patch results in a slow rise in blood levels and reaches its maximum after approximately 6 h (47). The prolonged presence of nicotine in case of patch application might thus explain the partially different effects of both applications.

Enhanced MEP-amplitudes after PAS-25 mimic LTP-like plasticity in transmitter-/receptor-involvement and time-course. LTP seems to be closely connected to memory function and cognitive performance. For these reasons, we draw a preliminary link between neurophysiologic and cognitive data obtained in this study and suggested that a relevant cause of nicotinic effects in deprived smokers on cognition might be the alteration of synaptic plasticity/activity and expression of LTP. Impaired general motor skill learning in deprived smokers [as compared to non-smokers (24)] might thus be due to reduced induction of excitatory LTP. Besides its impact on motor learning shown in this study, positive effects of nicotine in deprived smokers have already been described for working memory and attentional processes (48). It can thus not be ruled out that the improvement in general motor skill learning might at least partially depend on attentional processes.

Although plausible, these proposed mechanisms of action are largely speculative at present and need to be experimentally tested in future. Due to the complex impact of nicotine on a variety of neurotransmitters, neuromodulators, and different forms of nAChRs, alternative mechanisms cannot be ruled out.

### General Remarks

The results of the present study demonstrate that nicotine spray in deprived smokers influences both, LTP-like plasticity and motor learning performance. Nicotine withdrawal in smokers prevented the induction of PAS-25-induced excitatory neuroplasticity and nicotine administration restored absent plasticity and general motor learning skills in the SRTT-task. Our results are, therefore, in favor for the deficit-compensating hypothesis of nicotine consumption and matches previous observations where nicotine spray was able to normalize continuous attention, working memory, and computational processing in deprived smokers (49). Similar results have also been found in overnight nicotine-abstinent schizophrenic patients, where the administration of nicotine spray modestly enhanced attention and spatial working memory (50). In terms of nicotine addiction, our study indicates that cognitive impairment bases on nicotine deprivation and can be pharmacologically restituted. The effectiveness of treating nicotine dependency might be due to the fact, that current pharmacological treatments only address physical factors and miss out on the mental/cognitive factors. Our study suggests that the cognitive enhancing effect of nicotine may contribute to maintenance of nicotine addiction and needs to be addressed in treatment of nicotine addiction to avoid early relapse [see also Ref. (51)].

### Limiting Conditions

Some limitations of the present study should be taken into account. Although we matched the groups of the PAS-25 measurements and SRTT-performance in terms of age, gender, and working background, both studies were not performed in the same group. Thus, the connection of neurophysiologic results to cognitive functions is only indirect. Another limitation lies in the fact that we did not verify compliance of abstinence in smokers directly by obtaining nicotinic blood levels or breath CO. However, the Fagerstrom scale revealed only a light/moderate nicotine-dependent state; and with the beginning of the experiments in the morning (after a nicotine abstinent night), we relied on the statements of the subjects.

## Ethics Statement

The protocol was approved by the Ethikkommission der Universitätsmedizin Göttingen Von-Siebold-Str.3. The application number is 24/6/15. All subjects gave written informed consent in accordance with the Declaration of Helsinki.

## Author Contributions

JG and MN designed the study. NT, RA, and JG collected the data. JG and NT analyzed the data. MN and WP supervised the study. JG drafted the manuscript. All authors reviewed the manuscript.

## Conflict of Interest Statement

The authors declare that the research was conducted in the absence of any commercial or financial relationships that could be construed as a potential conflict of interest.
